# Dissecting Heterogeneous Populations of Protein-Complex Samples Using Direct Mass Technology

**DOI:** 10.1021/acs.analchem.5c05771

**Published:** 2025-12-02

**Authors:** Robert L. Rider, Jared Hampton, Zhenyu Xi, Carter Lantz, Sangho D. Yun, Weijing Liu, Rosa Viner, Arthur Laganowsky, David H. Russell

**Affiliations:** † Department of Chemistry, 14736 Texas A&M University, College Station, Texas 77843, United States; ‡ 10289 Thermo Fisher Scientific, San Jose, California 95134, United States

## Abstract

Charge detection mass spectrometry (CDMS) is an emerging technique for the qualitative analysis of heterogeneous biological samples by individually measuring the charge and *m*/*z* of ions to determine their mass. The commercialization of CDMS with Orbitrap-based CDMS has allowed for broader use of the technique within the native mass spectrometry (nMS) field. However, CDMS is still primarily used qualitatively, with only a few studies applying it for quantitation in the MDa range. Here, we demonstrate how Orbitrap-based CDMS/Direct Mass Technology (DMT) can be used for quantitation of heterogeneous transthyretin (TTR) tetramer samples <100 kDa. First, we demonstrate DMT can resolve wild-type (WT) and C-terminally tagged (CT) TTR homotetramers and hybrid tetramers (i.e., tetramers containing both types of subunits), enabling kinetic analysis of hybrid TTR formation. The CT-TTR homotetramer dissociates quicker, providing evidence that the C-terminal modification (-ASGENLFYQ) decreases the stability of the tetramer. We hypothesize this is due to altering important noncovalent interactions between subunits. Next, DMT is used to resolve and quantify thyroxine binding events to WT-TTR and CT-TTR, which can be utilized to determine binding affinity. Interestingly, CT-TTR has a higher affinity for thyroxine than WT-TTR, which indicates that the C-terminus is involved in TTR–ligand binding. In both examples, overlapping *m*/*z* signals that cannot be distinguished by conventional nMS are separated in CDMS through the additional charge measurement, enabling an accurate determination of mass and abundance. Overall, these results establish CDMS as a quantitative approach for heterogeneous protein samples and highlight its potential for broader applications in complex biological systems.

## Graphical abstract

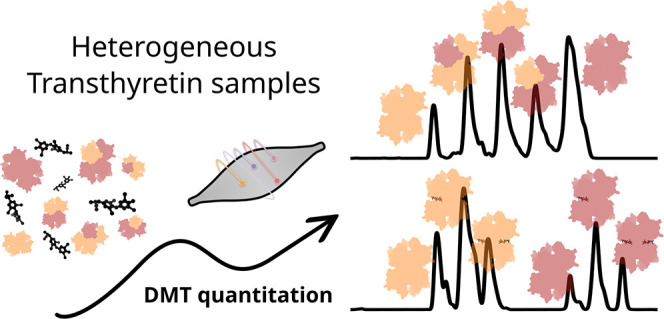

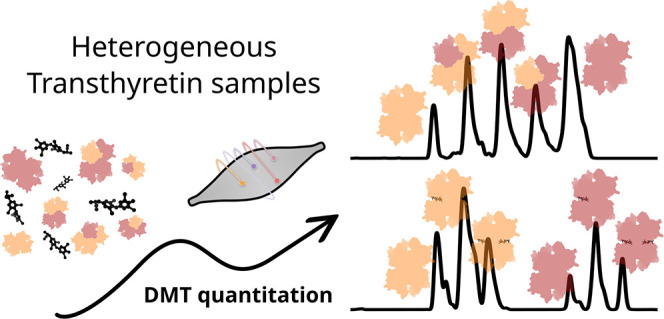

## Introduction

Transthyretin (TTR) is a homotetrameric transport protein that circulates in plasma and cerebrospinal fluid, delivering thyroxine (T_4_) and retinol-binding protein–vitamin A complexes.^1^ The stability of the TTR tetramer is central to its physiological role because dissociation into monomers can initiate misfolding and amyloid formation, leading to systemic or hereditary transthyretin amyloidosis.^2^ Subunit exchange between wild-type (WT) and variant TTR proteoforms, including disease-associated mutations such as V30M and V122I, have been implicated in amyloid progression by generating hybrid tetramers (tetramers containing WT and mutant subunits) with altered stabilities.^3,4^ Ligand binding, particularly at the two thyroxine-binding sites located at the dimer–dimer interface, can kinetically stabilize the tetramer and modulate amyloidogenicity.^5,6^ Accurate measurement of both subunit exchange kinetics and ligand binding stoichiometry is therefore critical for elucidating the TTR dynamics and stability.

Affinity or sequence tags are widely employed in protein expression, purification, or detection.^7,8^ While these tags are often assumed to be inert, their influence on protein structure and dynamics are rarely examined in detail.^9,10^ Protein tags at the N- and C-terminus of TTR have been shown to have differing gas phase stabilities from those of WT-TTR.^11^ The C-terminus is of particular interest because this region participates in the dimer–dimer interface that governs tetramer stability.^11,12^ Characterizing how the C-terminal tag (CT) (-ASGENLFYQ) affects TTR’s solution stability and ligand binding is not only relevant for understanding TTR dynamics but also provides an example of how expression tags can alter protein dynamics.^13^

Native mass spectrometry (nMS) has been invaluable for characterizing protein complexes,^14^ yet conventional nMS often struggles with heterogeneous samples where overlapping charge state distributions and adduct heterogeneity limit resolution and quantitation.^15^ Charge detection mass spectrometry (CDMS) addresses these limitations by independently measuring the charge and *m*/*z* of individual ions, enabling the direct determination of molecular mass and abundance. Development of CDMS has largely focused on achieving higher mass resolution^16^ and extending measurements toward assemblies into the GDa range.^17,18^ However, there is untapped utility for CDMS experiments other than mass and proteoform determination. Jordan et al. showed the capability to assess conformational heterogeneity of mAb aggregates using the added charge dimension that CDMS provides.^19^ The current work demonstrates that CDMS can separate proteoforms with overlapping *m*/*z* values based on charge and quantify their relative abundances in complex mixtures. While nMS is already a highly quantitative technique, our results establish CDMS as an equally powerful tool for quantitative analysis in heterogeneous systems.^20,21^

Here, we apply Orbitrap-based CDMS/Direct Mass Technology (DMT) using the selective temporal overview of resonant ions (STORI) approach^22,23^ to quantitatively compare WT-TTR and CT-TTR, focusing on two biologically relevant properties: tetramer stability assessed via subunit exchange kinetics and ligand-binding affinity. Conventional nMS cannot fully resolve the overlapping charge states of WT- and CT-TTR or their ligand-bound states. However, DMT can resolve these species by directly measuring the mass (*m*/*z* × charge), enabling quantitation of all proteoforms. The data reveal that the CT tag alters tetramer stability and thyroxine-binding affinity, demonstrating the structural sensitivity of TTR to modifications at its C-terminus. More broadly, this work demonstrates the impact that protein tags can have on native protein interactions and highlights the broader utility of quantitative CDMS for probing heterogeneous biological samples.

## Experimental Section

### Materials

Ammonium acetate, LC-MS grade water, LC-MS grade DMSO, pentetic acid (DTPA), l-thyroxine, and alcohol dehydrogenase were purchased from Millipore Sigma (Burlington, MA). Bio-Spin 6 kDa MWCO SEC desalting columns were purchased from Bio-Rad Laboratories (Hercules, CA) for buffer exchanges. WT-TTR and CT-TTR were expressed and purified as previously described.^4,13^ Custom nano-electrospray emitters were made using borosilicate glass and a Sutter Instruments P-1000 filament micropipette puller (Novato, CA). Each emitter was coated with gold using a Leica EM ACE200 sputter coater (Wetzlar, Germany).

### Sample Preparation

25 μL aliquots of WT- and CT-TTR were diluted in half with 1 M ammonium acetate and 3 mM DTPA and left to incubate for 30 min to remove zinc ions from expression. The samples were then buffer exchanged into 200 mM ammonium acetate. Concentrations of the protein sample were measured at 280 nm using an Eppendorf BioSpectrometer Basic (Hamburg, Germany). For subunit exchange experiments, 1 μM WT-TTR and CT-TTR were mixed together and left to incubate at 4 °C between time points to facilitate rapid dissociation and reassembly. Each replicate was done by using different samples of the same composition.

8.3 mg of thyroxine was dissolved in LC-MS grade DMSO and then diluted 100-fold in LC-MS grade water before adding it to TTR samples. 1 μM WT-TTR and CT-TTR were mixed together with 4 μM T_4_. Concentrations of T_4_ were originally tailored to show 0, 1, and 2 T_4_ bound states. Three replicates were completed with different samples of the same composition.

### Data Collection

All experiments were conducted on a Thermo Scientific Q-Exactive UHMR with added DMT capabilities (Bremen, Germany). Before DMT, mass spectra were collected at various resolving powers (6–200k) for each sample, with the instrument parameters reported in the Supporting Information (Table S1). For DMT experiments, the trapping gas pressure in the HCD cell was reduced to 0.3–0.8 to attenuate the signal intensity to a single ion level, as well as decreasing the UHV pressure to assist ion retention through the entire transient of Orbitrap analysis. We observed no significant difference between mass spectra collected at various HCD cell pressures (Supporting Information, Figure S1). For DMT collection, automated ion control (AIC) and 100% target density were used with 200k resolving power.

### DMT Post-Processing

The UHMR was initially calibrated for DMT low detector *m*/*z* target optimization and high ion transfer target *m*/*z* data collection using streptavidin (55 kDa), alcohol dehydrogenase (147 kDa), single-ring GroEL (400 kDa), and GroEL (801 kDa). DMT data were processed using the STORIboard software from Proteinaceous (Chicago, IL).^22^ The following are Voting v3 processing parameters: 0.996 r^2^ threshold, 0.42 duration threshold, 0.2 minimum time of death, 0.1 maximum time of birth, 3 S/N threshold, frequency correction is true, 3.5 bin size (ppm), minimum ions in bin are 1, 2 charge neighbors, and 10 isotope neighbors. Bin size was calculated using the largest mass observed, as found in eq 1 and rounded to the nearest half integer. DMT data were monitored in real time and collected until a minimum of 5000 ions was reached and 1000 ions were present in the most dominant proteoform based on the parameters noted above. Deconvoluted mass spectra from STORIboard were smoothed to a resolution of 0.008, where each proteoform was sufficiently resolved.

1 $$Bin\;size\;\left(ppm\right)=\frac{200,000}{Mass}=\frac{200,000}{61,200}=3.27\to 3.5$$

To extract abundance information, we put deconvoluted mass spectra into OriginPro (OriginLabs, Northampton, MA) and fit using the peak fitting tool. Each profile was fit with Gaussian’s using a first derivative search function and allowing the program to fit multiple peaks when proteoforms have adducted species. The area from the fit was used as the abundance of the proteoforms to determine mole fractions. Fitting for the kinetic plot and homotetramer dissociation rates was done with a first-order kinetic reaction exponential in MATLAB (Natick, MA).

## Results and Discussion

CDMS has emerged as a powerful tool for analyzing heterogeneous biomolecular samples, enabling accurate, direct mass determination by simultaneously measuring the charge and *m*/*z* for individual ions. While CDMS has been widely applied for qualitative analyses, its potential for quantitative and less than MDa applications remains underexplored. Figure 1A shows the theoretical mass spectrum of WT-TTR (14+ to 16+) and CT-TTR (15+ to 17+) homotetramers, where the two proteoforms become increasingly resolved as the charge states increase (<3900 *m*/*z*). Figure 1B zooms in on the 14+ (WT) and 15+ (CT) charge states, highlighting their strong overlap, which prevents accurate deconvolution of the proteoforms by conventional methods. Figure 1C displays theoretical distributions for 0, 1, and 2 T_4_ bound states of both WT- and CT-TTR tetramers, where <3900 *m*/*z* has observable separation between all proteoforms. However, for the 14+ and 15+ charge states of WT- and CT-TTR, respectively, the 0 T_4_- and 1 T_4_-bound populations overlap (Figure 1D). Furthermore, commonly adducted species (Na^+^, K^+^, and Zn^2+^) observed in experimental spectra broaden these distributions, making it increasingly difficult to distinguish and deconvolute the proteoforms (see Figure S2). Here, we employ Orbitrap-based CDMS/DMT to overcome this *m*/*z* overlap to resolve and quantify hybrid tetramer formation and T_4_ binding.

**Figure 1:**
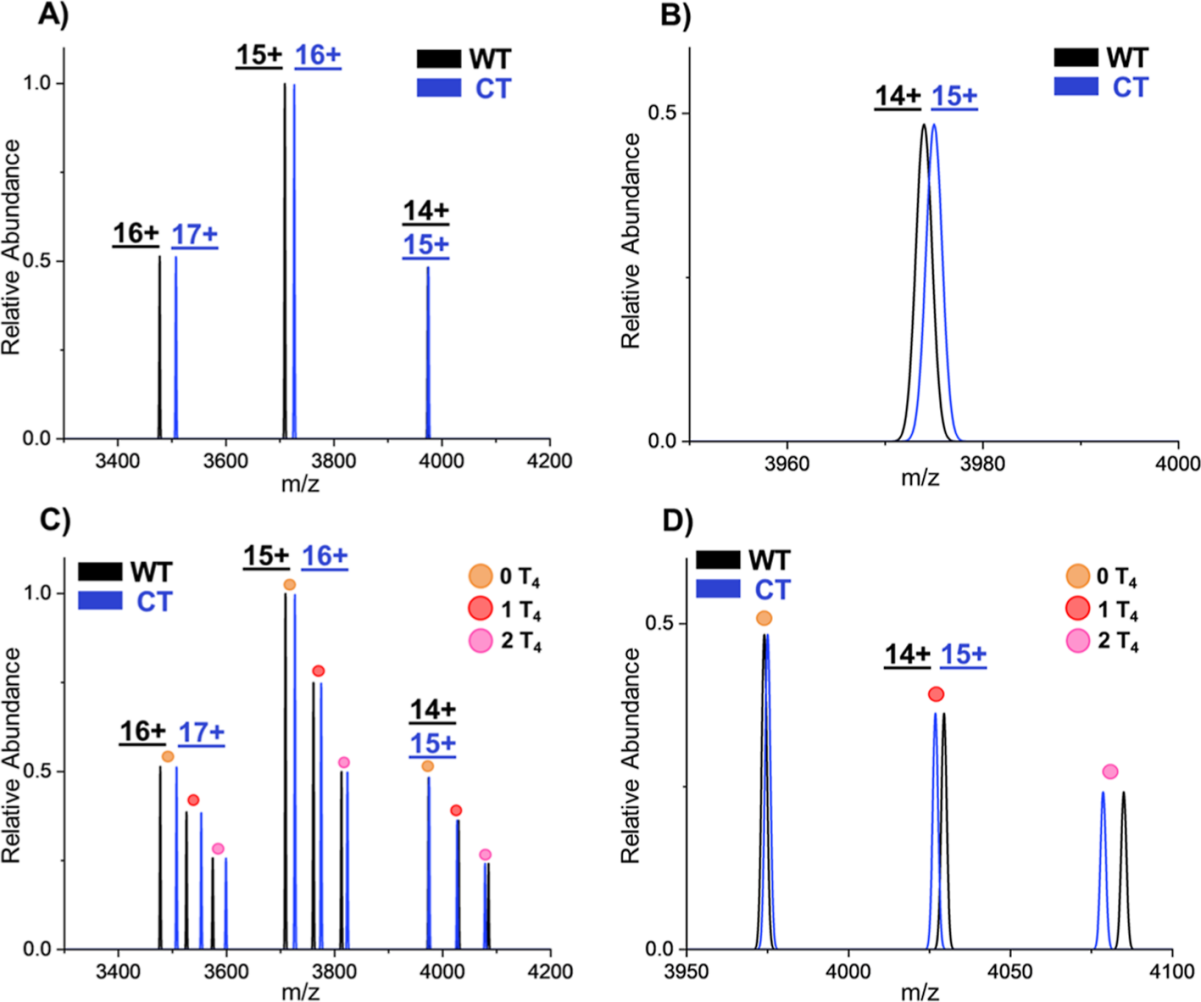
(A) Theoretical mass spectra of WT-TTR and CT-TTR homotetramers. (B) Zoomed-in view of the 14+ WT-TTR and 15+ CT-TTR distributions to show the large overlap of the proteoforms. (C) Theoretical mass spectra of WT-TTR and CT-TTR homotetramers with thyroxine (T_4_) binding. (D) Zoomed-in view of the 14+ WT-TTR and 15+ CT-TTR distributions where the 0 and 1 T_4_ have overlapping distributions. These theoretical distributions do not contain any commonly adducted species (Na^+^, K^+^, Zn^2+^), which broaden the distributions and create more overlap even for the proteoforms in the range 3300–3900 *m*/*z* (see Figure S2).

### WT-TTR and CT-TTR Subunit Exchange

WT-TTR naturally dissociates into monomers and dimers, which can reassemble into tetramers through the process of subunit exchange (SUE). Hybrid tetramers (i.e., tetramers containing both types of subunits) containing both WT and mutant subunits are biologically relevant as they have been implicated in amyloid progression due to altered stabilities.^24,25^ Previous nMS work using charge reducing buffers and ion mobility has confirmed additional dimer involved pathways that contribute to the production of hybrid states.^13^ Moreover, SUE is potentially important in the initial stages of TTR amyloidosis, where monomers produced from dissociation can misfold, initiating oligomer formation. To probe SUE of WT- and CT-TTR, without any solution- or gas-phase charge reduction, we used DMT, which can resolve the proteoforms that overlap in *m*/*z* but have different charges (Figure 2A,B).

**Figure 2:**
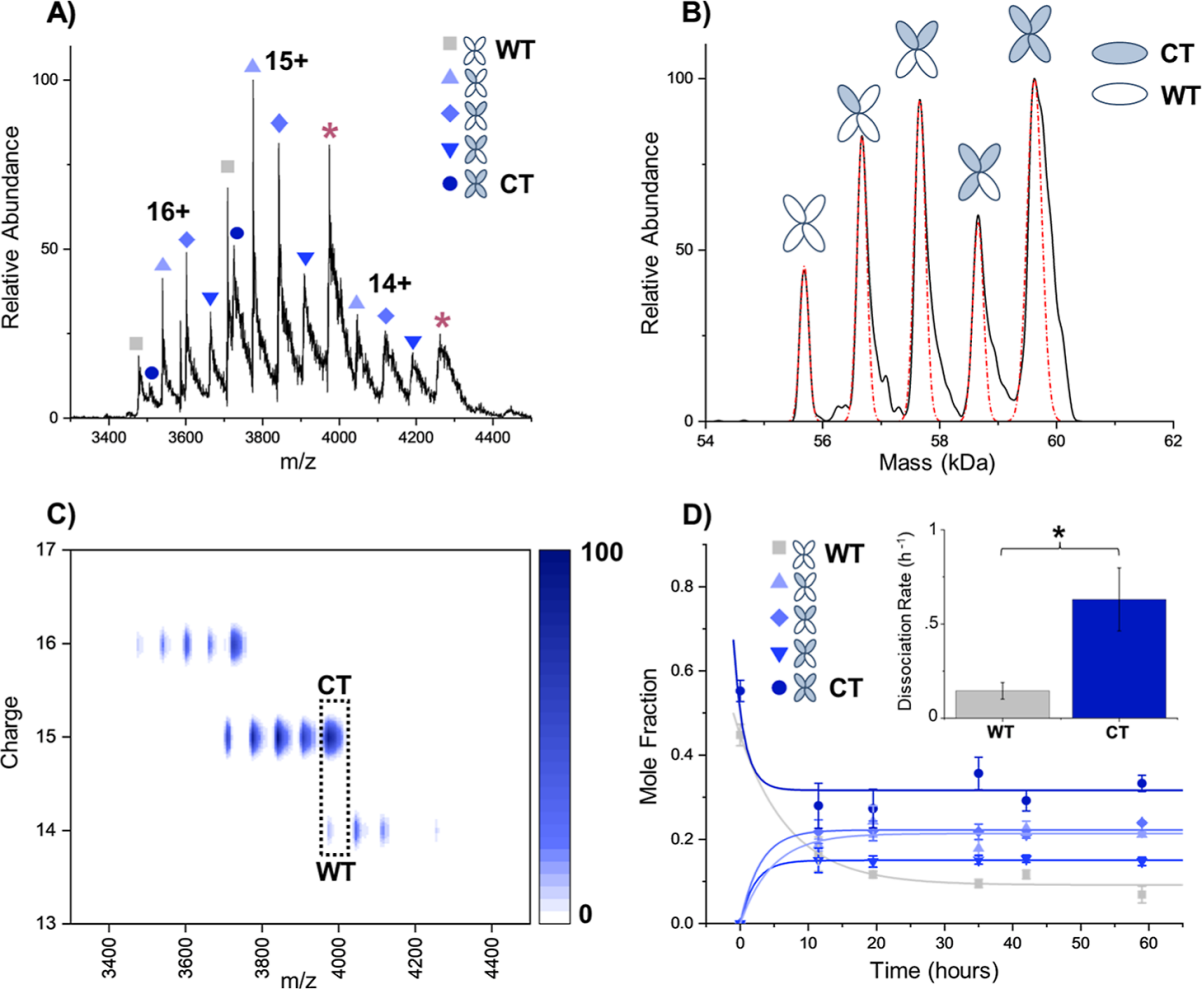
(A) Mass spectra of WT-TTR and CT-TTR subunit exchange after 35 h incubation at 4 °C. The hybrid tetramers are resolved and labeled; however, the homotetramers (*) cannot be fully resolved at each charge state. (B) Resultant deconvoluted mass spectrum from DMT analysis of the same sample. Each TTR proteoform is resolved, and fit for abundance analysis is shown in red. Tetramers containing CT-TTR subunits hold onto more adducts, leading to a wider right side of the mass peaks. (C) 2D heatmap of the *m*/*z* vs charge, which displays the separation of homotetramers achieved by the DMT measurement at ∼4000 *m*/*z*. (D) Subunit exchange kinetics plot derived from DMT data. The inset contains the dissociation rate quantitation for WT- and CT-TTR homotetramers. Error bars represent the standard deviation of replicates (*n* = 3). *p < 0.05.

Figure 2A illustrates the mass spectrum of WT- and CT-TTR subunit exchange following a 35 h incubation at 4 °C. Although hybrid tetramers are distinguishable, homotetramers remain unresolved at ∼4000 and 4300 *m*/*z*, limiting direct quantitation from raw mass spectra. However, using DMT analysis (Figure 2B), all 5 proteoforms (2 homo and 3 hybrid tetramers) are resolved. A clear depiction of how DMT resolves the homotetramers at ∼4000 *m*/*z* due to the added charge measurement is seen in the 2D *m*/*z* versus charge plot (Figure 2C). Figure S3 shows highly similar mass spectra before and after STORI processing, demonstrating DMT’s capability to resolve (*z* and *z* + 1) ions of the homotetramers at the same *m*/*z*. No charge-state- or *m*/*z*-dependent bias was observed, which we attribute to the sufficient ion charge (13 to 17+) and single-ion detection inherent to the method. Notably, CT-TTR exhibits a broader mass distribution due to the retention of adducted species and truncated oxygen species (see the Supporting Information, Figure S2). As a result, the hybrid tetramer distributions in Figure 2B also retain adducts proportional to the amount of CT character that makes them up. Therefore, to get peak abundances, a Gaussian curve was fitted to each proteoform, and peak area was used for analysis.

Kinetic analysis of SUE reveals the growth of the hybrid states over time and distinct dissociation rates for WT- and CT-TTR homotetramers (Figure 2D). As time progresses, the production of hybrid tetramers at 4 °C increases, reaching equilibrium after around 35 h. The hybrid tetramer containing 1 WT- and 3 CT-TTR subunits has the lowest abundance throughout the time points and does not approach abundance toward the 3 WT- and 1 CT-TTR hybrid tetramer as seen previously.^3,13,26^ Due to the presence of various proteoforms and adducted species for CT-TTR (see Supporting Information, Figure S2), it is possible the concentration of CT was less than that of WT, resulting in the preference to produce more of the 3 WT and 1 CT hybrid tetramer than the 1 WT and 3 CT hybrid tetramer (Figure 2B). Additionally, it is possible the broadened CT mass peak seen in Figure 2B may need to be fit with more constraints (i.e., set FWHM and number of Gaussians) to capture the potentially lower abundance of CT. Regardless, since the mass data were fit consistently across all spectra collected, the dissociation rates (Figure 2D) would not change.

To determine the stability of the homotetramers, the dissociation rates were extracted by fitting the data to a first-order exponential decay shown in Figure 2D. The observed dissociation rates of 0.145 h^–1^ and 0.630 h^–1^ for WT- and CT-TTR, respectively, are consistent with previous measurements.^13^ The data suggest that CT-TTR tetramers exhibit altered stability relative to WT, likely due to differences in solvent accessibility or electrostatic interactions at the C-terminus. Previously, we have shown that the C-terminal tag does not alter the TTR gas-phase stability as much as the N-terminal dual flag tag that was used extensively when studying TTR SUE.^11^ However, we acknowledge that solution- and gas-phase stabilities are different, and the SUE dissociation rate is a more accurate measure of solution stability than the previous CIU experiments.^27–29^ Extremely stable (T119M)^30^ and unstable (V122I)^25^ TTR mutants are found right at the C-terminus and are involved in the dimer–dimer interface.^12^ In conjunction with CT-TTR being more unstable than WT-TTR, this work indicates the importance of the noncovalent interactions at the C-terminus for tetramer stability. Importantly, these results demonstrate that DMT can be employed to extract quantitative kinetic information about protein–protein interactions in heterogeneous samples.

### Thyroxine Binding to TTR Homotetramers

Thyroxine is the less active form of a thyroid hormone important for metabolism that binds to TTR for transport throughout plasma and cerebral spinal fluid.^31^ Turnover of T_4_ into T_3_ (active) occurs after dissociation, where it can then modulate gene expression via binding to nuclear receptors.^32^ The TTR tetramer gains substantial stability as it binds up to 2 T_4_ at the tetramer interface, decreasing tetramer dissociation and SUE.^33^ This has been shown previously to decrease the amyloidogenic rate of TTR, which led to tafamidis (similar in structure to T_4_) to be designed and is currently the only drug that is approved for clinical treatment of TTR amyloidosis.^34^ Measuring the difference in binding affinity between TTR proteoforms may provide insight into regions of TTR or TTR intermolecular/intramolecular interactions that are important for binding of small molecules such as tafamidis and T_4_.

DMT was further applied to quantify T_4_ binding to WT- and CT-TTR homotetramers, highlighting the technique’s utility in probing protein–ligand interactions. Figure 3A shows a representative mass spectrum of TTR homotetramers in the presence of T_4_. While ligand-bound species can be observed, homotetramers cannot be confidently resolved across the entire 3000–5000 *m*/*z* range. By leveraging the charge-resolving capabilities of DMT, we achieve separation of the homotetramers (Figure 3B,C) and the 0, 1, and 2 ligand-bound states. The 2D plot clearly shows the gain in resolution achieved when charge is simultaneously measured with *m*/*z* by separating the 14+ and 15+ charge states for WT and CT, respectively, in the range 3900–4200 *m*/*z*. Additionally, using ion mobility does not provide the same kind of 2D resolution due to the similarity in arrival times for the homotetramer proteoforms, as evidenced by Figures S4 and S5 (see the Supporting Information). These data provide evidence that when ion mobility is able to separate out species that overlap in *m*/*z* yet differ in mobility, CDMS could be an alternative or used complementarily since the species likely also differ in charge.

**Figure 3:**
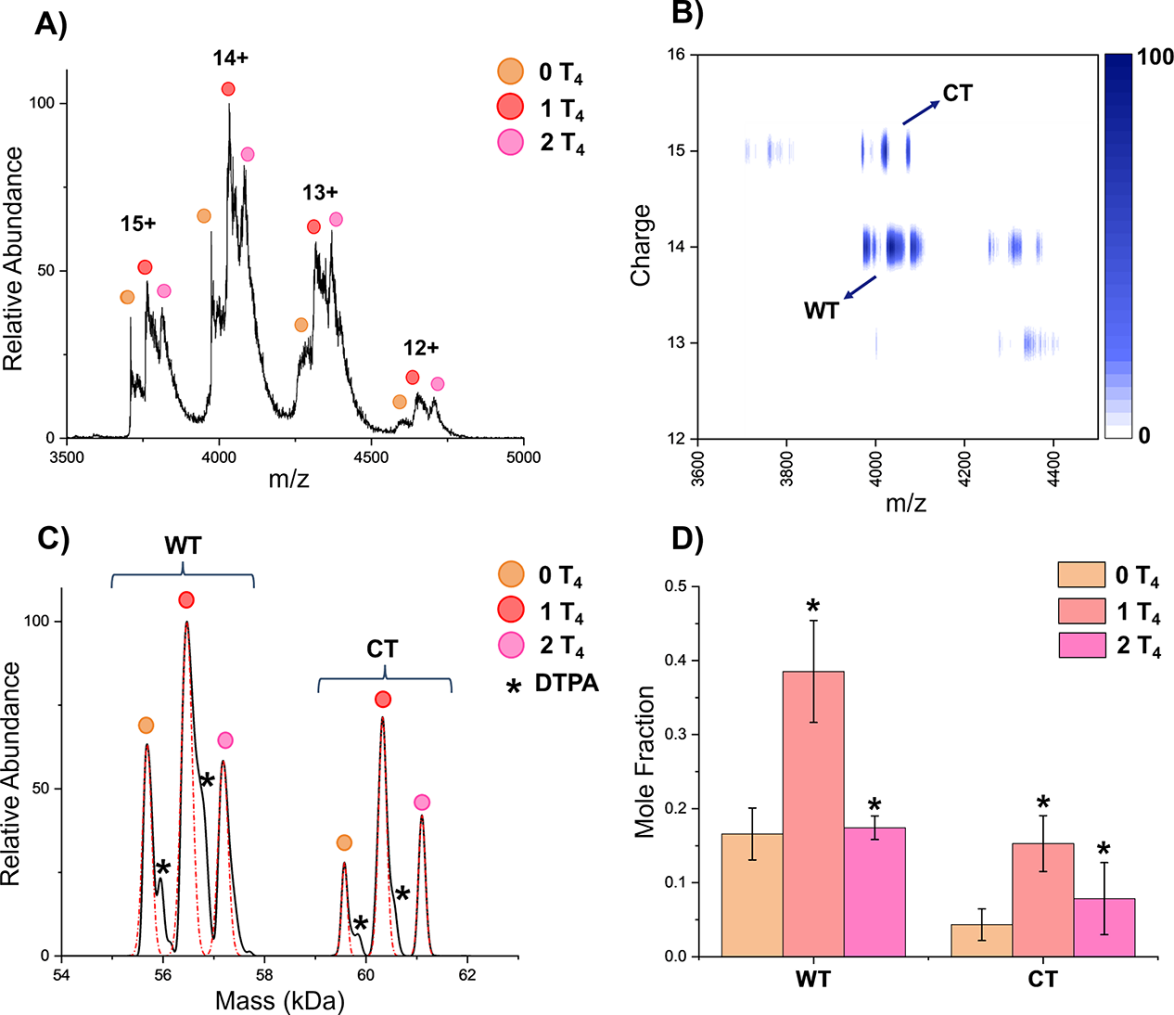
(A) Mass spectra of WT-TTR and CT-TTR homotetramers with thyroxine binding. Ligand binding can be resolved, but homotetramers cannot be confidently identified. Charge states labeled are for WT; CT is always one charge state higher at the same *m*/*z* range. (B) DMT heatmap resolving WT-TTR and CT-TTR homotetramers with the additional measurement of charge. (C) Deconvoluted mass spectrum from DMT analysis. Both homotetramers and their ligand binding are resolved, and fit for analysis is shown in red. (D) Mole fraction of each homotetramer and their thyroxine binding species. Error bars represent the standard deviation of replicates (*n* = 3). The significance for each ligand state is in respect to the left adjacent state. *p < 0.05.

DMT data (Figure 3C) reveal resolved 0, 1, and 2 thyroxine binding distributions for WT- and CT-TTR homotetramers even when the proteoforms were not resolved in the mass spectrum. The quantitation of bound and unbound species (Figure 3D) indicates a significant difference between each adjacent TTR-T_4_ state. Additionally, Figure S6 (see the Supporting Information) shows that the binding affinity for CT-TTR is slightly higher than that for WT-TTR with 1.11 and 1.01 T_4_ bound on average, respectively. The increased binding preference of CT-TTR suggests that modifications at the C-terminus alter thyroxine interaction(s), potentially through allosteric effects or changes in the thyroxine-binding pocket dynamics.^35^ These findings underscore the utility of CDMS for quantifying ligand binding events accurately.

As noted above, there are various mutants on the C-terminal end that cause drastic changes to TTR tetramer stability, as well as T_4_ binding affinities.^12^ T119M is more stable than WT and has a higher T_4_ binding affinity, while V122I is less stable and has a lower binding affinity than WT.^25,30^ CT-TTR has a lower tetramer stability (see Figure 2D) yet a higher T_4_ binding affinity (see Supporting Information Figure S6). We theorize this is due to the increasing complexity of interactions when you add 9 amino acids in CT-TTR’s case versus changing one for the mutants listed above.^36^ The C-terminal tag potentially interrupts important intermolecular interactions about the dimer–dimer (tetramer) interface, resulting in quicker tetramer dissociation.^12^ However, it may also open up the interfacial region more to allow for more favorable binding of T_4_.^37,38^ Additionally, there are known important structurally stabilizing (cold) water molecules near the C-terminus and tetramer interface that may be perturbed due to changes in protein hydration about the C-terminal tag.^39,40^ This could result in decreased tetramer stability yet higher affinity for T_4_, as there is less entropic penalty to remove the water molecules from the binding domain to allow T_4_ binding.^41,42^

The ability of DMT to resolve overlapping *m*/*z* species by charge provides a unique advantage in quantifying heterogeneous protein states, particularly in systems where conventional native mass spectrometry lacks the resolving power to distinguish closely spaced species in *m*/*z*.^4^ Other methods such as ultrahigh-resolution FTICR, electron capture charge reduction, and proton transfer charge reduction offer higher throughput analysis and can also identify the abundance of proteoforms with small mass differences.^43–46^ However, due to the breadth of utility that the UHMR has for protein (kDa to MDa) analysis,^47^ DMT is a promising technique for resolving heterogeneous samples without instrument modification.^48^ Additionally, charge-reducing buffers have been shown to aid in resolving condensed heterogeneous mass spectra.^49^ However, previous work with GroEL-ATP binding provides evidence that protein interactions are not always the same in ethylene diamine diacetate (EDDA) as in ammonium acetate.^50,51^ Therefore, the use of EDDA and triethylamine acetate requires careful consideration due to potential impacts on the biological system.

The application of DMT to monitor TTR subunit exchange and ligand binding demonstrates its potential for investigating protein stability and interactions induced by protein modifications. Given the role of TTR in amyloid diseases, the quantitation of differences between WT- and CT-TTR tetramer dissociation and ligand binding has indicated that the C-terminus plays a role in TTR interactions and stability more than previously thought. Most structural work negates the dynamic N- and C-termini as they are disordered loop regions that are unresolved in X-ray crystallography and cryo-EM imaging, making it difficult to draw conclusions on their effects in TTR dynamics.^37,52,53^

## Conclusion

This work demonstrates that quantitatively, Orbitrap-based CDMS/DMT can determine differences in protein-complex stability and ligand binding. By directly comparing WT- with CT-TTR, we show that the CT tag accelerates tetramer dissociation while enhancing T_4_ binding affinity. These results indicate that the C-terminus, located at the dimer–dimer interface, plays a more significant role in TTR stability and ligand recognition than previously appreciated.^12^ Even the addition of a short peptide sequence perturbs noncovalent interactions at the interface, likely altering solvent accessibility, and the dynamics of the thyroxine binding channel.^37,54^ Such findings underscore the importance of evaluating how seemingly minor sequence modifications, such as affinity tags, can influence fundamental biophysical properties.

Beyond TTR, this work positions quantitative CDMS as a broadly applicable strategy for analyzing heterogeneous protein systems, where charge domain separation overcomes the resolution limits of *m*/*z* domain measurements in traditional nMS experiments. While much of the field has emphasized extending CDMS to ultrahigh masses and maximal resolution,^16,18^ our results highlight the underexplored potential of applying the method in the 1000–10,000 *m*/*z* range common to nMS experiments. This capability is directly relevant to heterogeneous membrane protein complexes, which often display broad distributions of oligomeric states, PTMs, lipid, and ligand adducts that complicate spectra.^48,55^ Similarly, mAbs can oligomerize and exhibit mass heterogeneity from variable glycosylation, affecting pharmacokinetics, effector function, and stability.^19^ CDMS may resolve and quantify such proteoforms in a single experiment without the need for charge reduction or chemical derivatization.^48,56,57^

## Supplementary Material



## Notes

Protein sequences, instrument parameters, comparison of trapping gas pressures, and ion mobility mass spectrometry separation (PDF)
