# Supplementary material for: Dissecting Heterogeneous Populations of Protein-Complex Samples Using Direct Mass Technology
Source: Anal Chem. 2025 Dec 2;97(49):27057–63. doi: 10.1021/acs.analchem.5c05771 (PMC12713611; doi:10.1021/acs.analchem.5c05771)
Supplement: Supplementary file 1 [file ac5c05771_si_001.pdf]

Supporting Information

Dissecting Heterogeneous Populations of Protein-Complex Samples Using Direct Mass Technology

Robert L. Rider[1], Jared Hampton[1], Zhenyu Xi[1] ,Carter Lantz[1], Sangho D. Yun[1], Weijing Liu[2], Rosa Viner[2], Arthur Laganowsky[1], David H. Russell[1]*

[1]Texas A&M University Department of Chemistry, College Station TX, 77843

[2]Thermo Fisher Scientific, San Jose CA, 95134

*Corresponding author: russell@chem.tamu.edu

Table of Contents

	Page
Experimental	S2
Figure S1.	S3
Figure S2.	S4
Figure S3.	S5
Figure S4.	S6
Figure S5.	S7
Figure S6.	S8
Table S1.	S9
References	S10

Experimental

The following are the monomer sequences of the recombinantly expressed TTR used in the manuscript. The amino acids in bold were left from the TEV protease recognition site for protein purification on the N-terminus of WT-TTR.[1] The amino acids italicized indicate the C-terminal tag on CT-TTR.[2] This tag consists of the tripeptide 'ASG' which links the TTR C-terminus and the first six residues of the TEV protease recognition sequence remaining after cleavage.

WT-TTR Sequence:

GSGPT GTGES KCPLM VKVLD AVRGS PAINV AVHVF RKAAD DTWEP FASGK TSESG ELHGL TTEEE FVEGI YKVEI DTKSY WKALG ISPFH EHAEV VFTAN DSGPR RYTIA ALLSP YSYST TAVVT NPKE

Tetramer Molecular Weight (avg): 55622.16

pI: 5.31

CT-TTR Sequence:

MGPT GTGES KCPLM VKVLD AVRGS PAINV AVHVF RKAAD DTWEP FASGK TSESG ELHGL TTEEE FVEGI YKVEI DTKSY WKALG ISPFH EHAEV VFTAN DSGPR RYTIA ALLSP YSYST TAVVT NPKE*A SGENL FYQ*

Tetramer Molecular Weight (avg): 59610.68

pI: 5.15

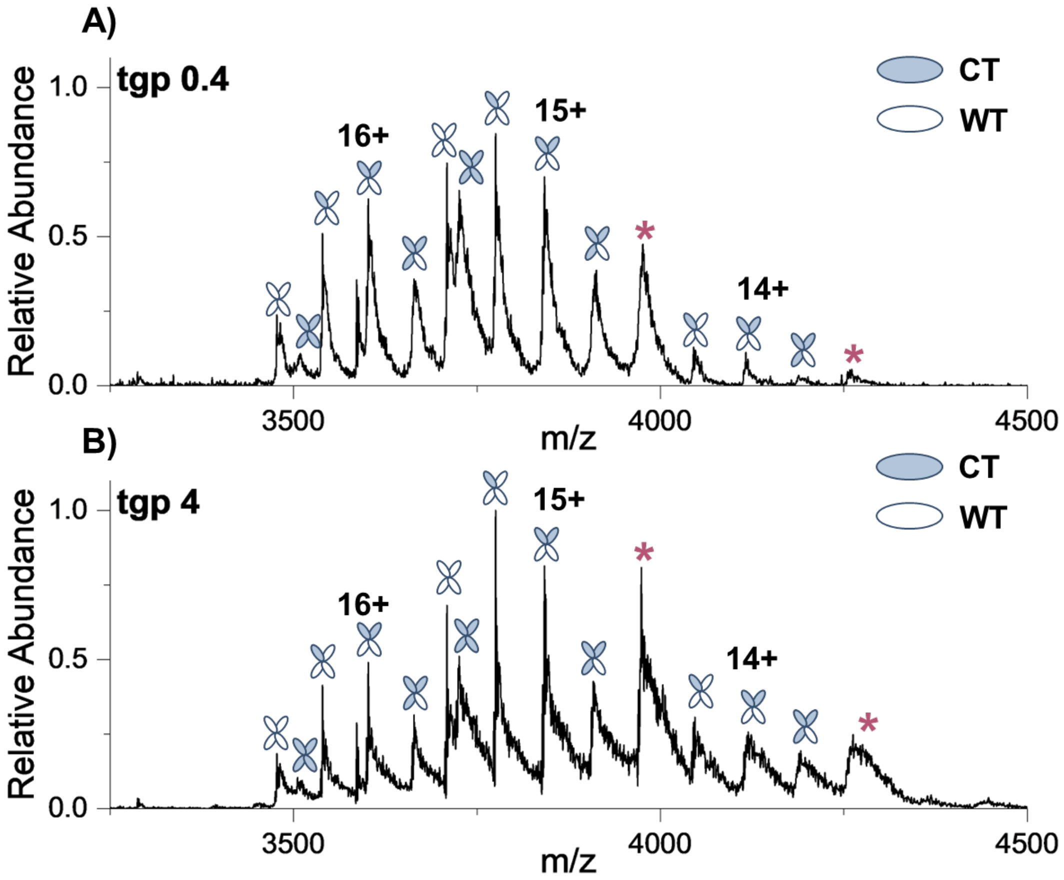

Figure S1. Mass spectra of WT- and CT-TTR homotetramer subunit exchange in 200 mM AA after 35 h at 4 °C. Charge states are labeled for the hybrid tetramers and the asterisk (*) denotes unresolved homotetramer peaks. **A)** Spectra collected at 0.4 trapping gas pressure (tgp) and **B)** 4 tgp in the UHMR HCD cell. All CDMS data was collected around 0.3-0.8 tgp to attenuate the signal to the single ion level, while regular nMS collection was done at 4 tgp. There is no significant difference between the resolution of the proteoforms and the abundance of the species, providing evidence CDMS data can be collected by attenuating signal via the HCD cell pressure. When samples being measured span a wider m/z, charge and/or mass range, there may be more differences observed for the mass spectra and thus CDMS results when changing the HCD cell pressure.

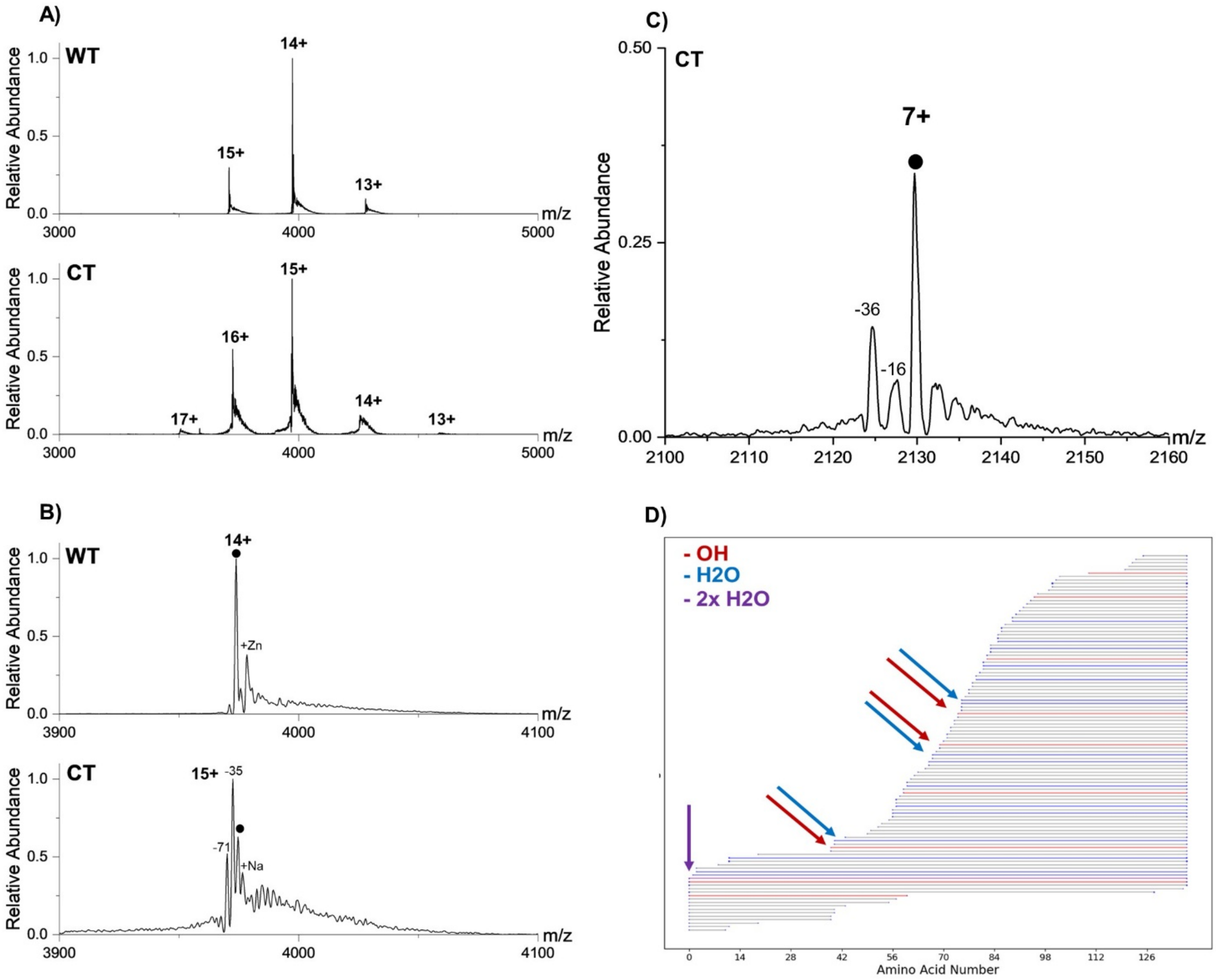

Figure S2. A) Mass spectrum of WT- and CT-TTR homotetramers in 200 mM AA. CT has on average 1 charge higher than WT and retains more adducted species as seen with the increased tailing on the right side of the charge states. **B)** Enlarged view of the charge state found at ~3975 m/z for both homotetramers where the theoretical mass is labeled with the black dot. WT only retains a minor amount of zinc, while CT has an abundance of adducts and a few species to the left of the theoretical mass. **C)** Magnified view of mass spectrum of CT TTR monomer 7+ after collision induced dissociation with the theoretical mass labeled with the black dot. CT has two prominent peaks on the left side. **D)** Top-down analysis of CT using ClipsMS to search for unlocalized modifications with < 3 ppm error.[3] Gray lines represent non-modified b and y fragment ions, while the colored lines indicate various different oxygen modifications. There are fragments that align with -OH and -H_2O for the approximate -16 Da species and -2x H_2O for the -36 Da species. Arrows are used to show modified terminal fragment ions that have a non-modified counterpart. The truncation of CT via oxygen products is believed to be the result of long-term storage at -80 °C after expression.

\

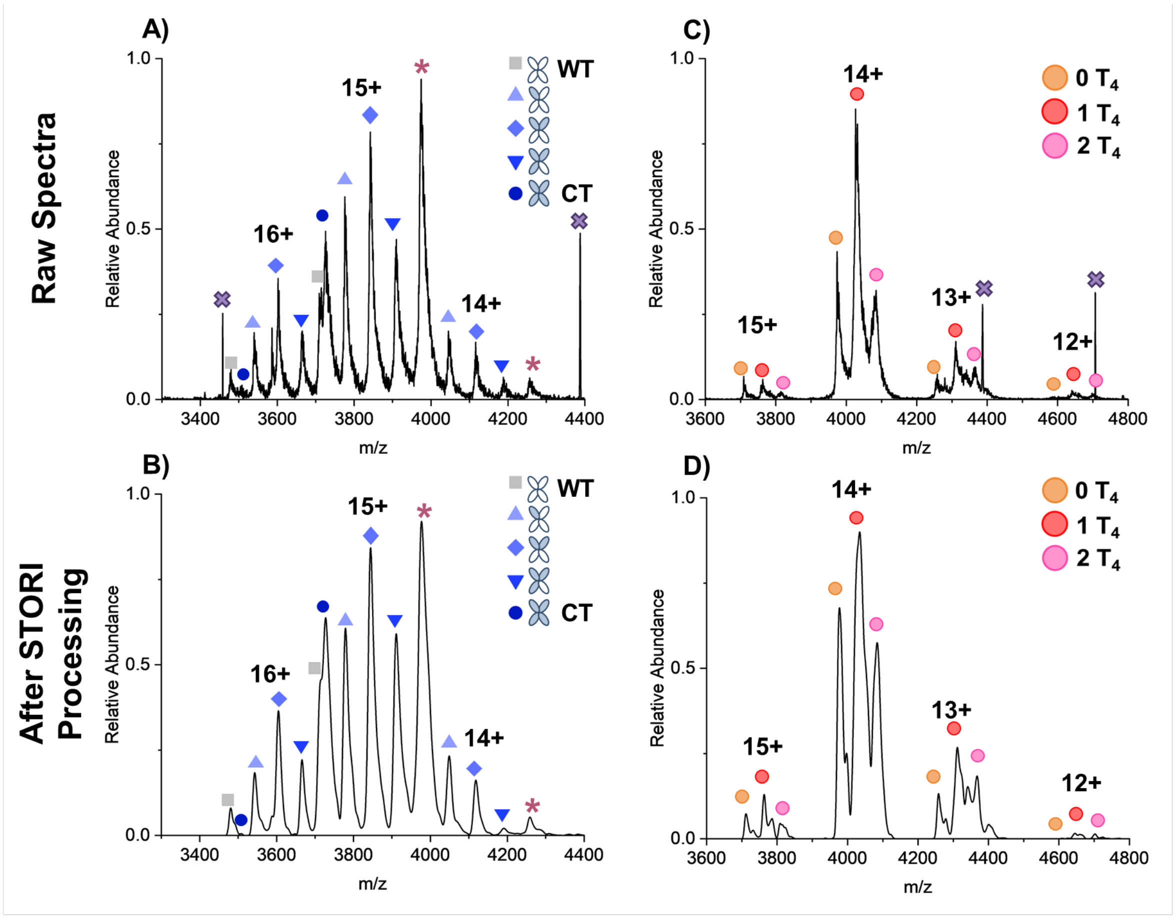

Figure S3. A) Mass spectrum of WT-TTR and CT-TTR subunit exchange after 35 h incubation at 4°C collected at 200k resolving power with DMT mode on. The hybrid tetramers are resolved and labeled, however the homotetramers (*) cannot be fully resolved at each charge state. **B)** Mass spectrum of the same subunit exchange data as in **A)** after it was processed with STORIboard. **C)** Mass spectrum of WT-TTR and CT-TTR thyroxine binding collected at 200k resolving power with DMT mode on. Ligand binding can be resolved, but homotetramers are not. Charge states labeled are for WT; CT is always one charge state higher at the same m/z range **D)** Mass spectrum of the same ligand data as in **C)** after it was processed with STORIboard. There is no observable difference in proteoform relative abundance after the data is processed with STORIboard, indicating that there is no bias induced by processing and m/z overlap of the WT- and CT-TTR proteoforms at ~3900 and ~4300 m/z or with the ligand binding. Purple (**X**) as seen in **A)** and **C)** pertains to instrument noise, which is filtered out with STORIboard processing.

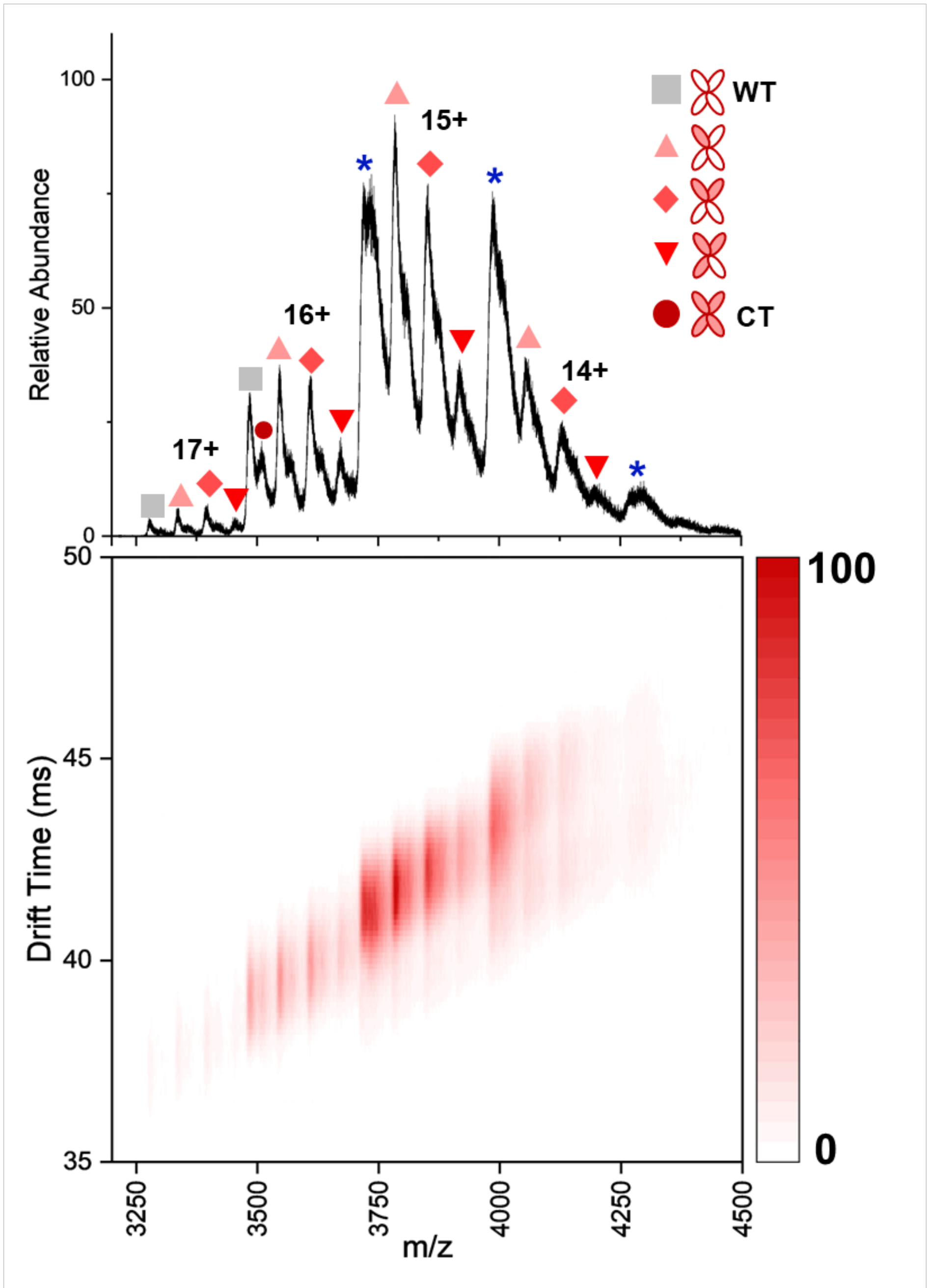

Figure S4. WT- and CT-TTR subunit exchange ion mobility data collected on an Agilent 6560 IM-Q-TOF using nESI. Charge states labeled are for WT and hybrid tetramers; CT is always one charge state higher at the same m/z range. The mass spectrum shows the hybrid tetramers are well resolved, yet the homotetramers are not well resolved at each charge state, indicated by the asterisk (*). The 2D plot of m/z versus drift time does not provide any additional degree of separation for WT and CT homotetramers due to the similar arrival times despite their different charges.

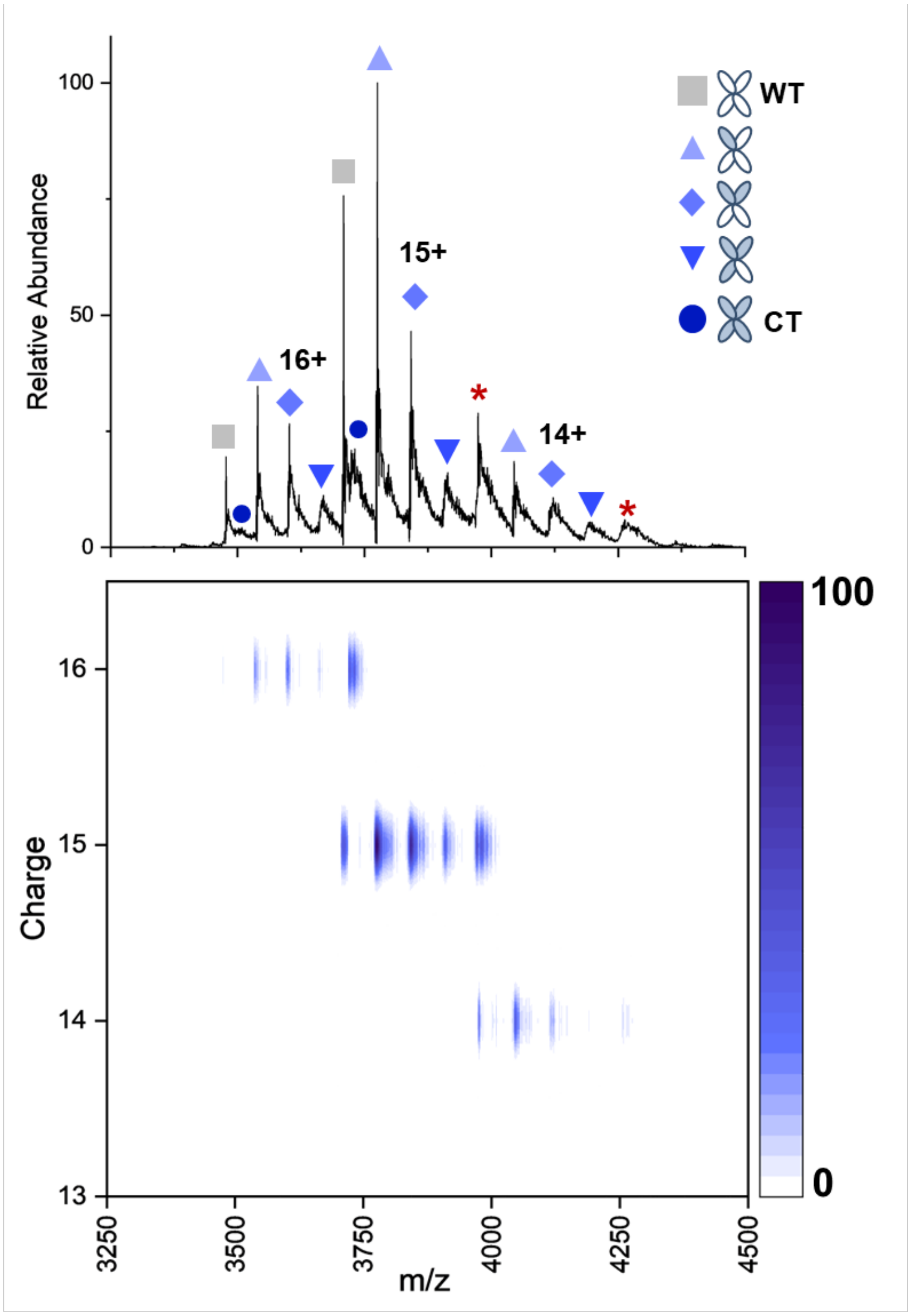

Figure S5. WT- and CT-TTR subunit exchange data collected in regular mode and the resultant CDMS 2D plot with the ThermoFisher UHMR. Charge states labeled are for WT and hybrid tetramers; CT is always one charge state higher at the same m/z range. The mass spectrum shows the hybrid tetramers are well resolved, yet the homotetramers are not well resolved at each charge state, indicated by the asterisk (*). The 2D plot of m/z versus charge provides excellent resolution of each TTR proteoform, especially for WT- and CT-TTR homotetramers due to their overlapping in m/z but different charge.

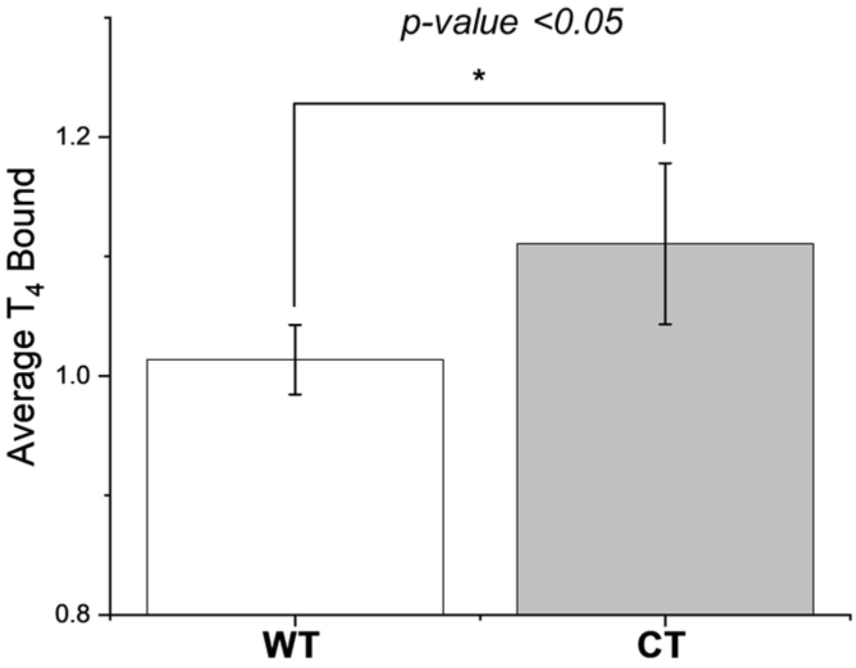

Figure S6. WT- and CT-TTR T_4 binding affinity within the same solution. Average T_4 bound was calculated using the relative abundances of the 0,1 and 2 T_4. CT TTR has a stronger affinity for T_4, which may be due to the differences in inter- or intramolecular interactions with the addition of the C-terminal tag (-ASGENLFYQ). Error bars represent standard deviation of replicates (n=3).

Table S1. ThermoFisher Q-Exactive UHMR Parameters for TTR Measurements

UHMR Parameters	Regular	CDMS
Spray Voltage (kV)	1.0 – 1.7	1.0 – 1.7
Capillary Temperature	100	100
Source DC Offset (V)	21	21
In Source Trapping	On	On
Desolvation Voltage (V)	-10	-10
In Source CID (eV)	50	50
HCD CE	50	50
Trapping Gas Pressure Setting	4	0.3-0.8
Injection Flatapole DC (V)	8	8
Inter Flatapole Lens (V)	4	4
Bent Flatapole DC (V)	3	3
Transfer Multipole DC (V)	0	0
Detector m/z Target Optimization	Low m/z	Low m/z
Ion Transfer Target m/z	High m/z	High m/z
Sample Collection Time (min)	1	15-40

References:

(1) Lantz, C.; Rider, R. L.; Yun, S. D.; Laganowsky, A.; Russell, D. H. Water Plays Key Roles in Stabilities of Wild Type and Mutant Transthyretin Complexes. *Journal of the American Society for Mass Spectrometry* **2024**, *35* (8), 1854–1864. DOI: 10.1021/jasms.4c00170.
(2) Shirzadeh, M.; Boone, C. D.; Laganowsky, A.; Russell, D. H. Topological Analysis of Transthyretin Disassembly Mechanism: Surface-Induced Dissociation Reveals Hidden Reaction Pathways. *Anal Chem* **2019**, *91* (3), 2345–2351. DOI: 10.1021/acs.analchem.8b05066 From NLM Medline.
(3) Lantz, C.; Zenaidee, M. A.; Wei, B.; Hemminger, Z.; Ogorzalek Loo, R. R.; Loo, J. A. ClipsMS: An Algorithm for Analyzing Internal Fragments Resulting from Top-Down Mass Spectrometry. *Journal of Proteome Research* **2021**, *20* (4), 1928–1935. DOI: 10.1021/acs.jproteome.0c00952.
